# Exploring Environmental Considerations for Terminally Ill Pediatric Patients in Palliative Care Inpatient Units: A Narrative Review

**DOI:** 10.1177/19375867241271439

**Published:** 2024-08-19

**Authors:** Sara Nourmusavi Nasab, Rebecca McLaughlan, Chris L. Smith

**Affiliations:** 1 School of Architecture, Design and Planning, 4334 The University of Sydney, Sydney, Australia

**Keywords:** narrative review, terminally ill, pediatric patients, palliative care, built environment

## Abstract

**Background:** The end-of-life experience is significantly influenced by the surrounding environment, emphasizing the importance of exploring built environmental factors in palliative care, especially for pediatric patients. As the majority of end-of-life individuals are elderly or adults, most studies have focused on the environment for this demographic. However, it is essential to recognize that children and adolescents may have distinct needs in this regard. **Aim:** This narrative review aims to explore the impact of the built environment on pediatric end-of-life patients in inpatient units within palliative care settings. **Method:** A comprehensive search was conducted across four key databases (PubMed, MEDLINE, PsycINFO, and CINAHL) to identify relevant articles. The screening process commenced with an initial assessment of article titles and abstracts, followed by a thorough examination of full-text studies that met the inclusion criteria. Data synthesis involved thematic analysis facilitated by NVIVO software and informed by the findings extracted from selected literature. **Results:** The review identified 22 studies meeting inclusion criteria, revealing key insights into environmental considerations in pediatric palliative care. Four themes emerged, highlighting the significance of activities and play environments, accommodation spaces for patients, supportive spaces for families, and outdoor and green spaces. **Conclusions:** Acknowledging limited research on architectural aspects and reliance on family and staff perspectives, future studies should prioritize understanding pediatric patients’ perspectives, particularly adolescents. The study underscores the importance of enhancing environmental design in pediatric palliative care to meet the unique needs of patients and their families.

## Introduction

The place where end-of-life patients reside and receive care until their death becomes vital, significantly impacting their comfort and overall quality of life (Pinto et al., 2023). Just as we have a unique bond with our homes, it is common for individuals to prefer receiving end-of-life care in their residences (Campbell, 2020). Despite the preference for alternative settings, such as home or hospice care, most deaths occur in medical settings like hospitals (Broad et al., 2013). This trend is also evident among pediatric patients in Australia (Australian Bureau of Statistics, 2021). Recognizing that a person's location at the time of death is a significant factor in determining the quality of an end-of-life experience, it becomes imperative to explore physical, environmental, and architectural considerations that can be beneficial in delivering supportive end-of-life care. This is particularly important for pediatric patients, including children and adolescents, who may have specific environmental requirements. Advances in medical care coupled with the earlier recognition of life-limiting diagnoses, have increased the number of children, adolescents, and young adults, living with life-limiting conditions (Lin et al., 2021); and it is important to recognize that this age group presents distinct medical, psychological, social, and supportive care requirements that set them apart from their adult counterparts (Clark & Fasciano, 2015). These distinctions are particularly noticeable in pediatric care, emphasizing the necessity for customized approaches to meet the specific needs of various age groups. This narrative review aims to explore how the built environment influences pediatric palliative care, identifying spaces, features, or qualities that can positively or negatively impact the experience for children and/or adolescent patients, their families, and staff.

### Method

This narrative review utilizes a systematic sampling strategy, starting with a thorough exploration of literature across four databases (PubMed, MEDLINE, PsycINFO, and CINAHL). The search focused on articles discussing the physical environment's needs of pediatric patients and families in palliative care facilities. Employing Boolean operators, the search terms included Physical environment OR Healthcare design OR Architecture/End-of-life OR palliative OR terminal/Pediatric OR children OR adolescents OR youth OR Teenager/ AND 2 AND 3. The review was further enriched by a snowballing process, manually examining reviews for relevant articles known to the authors. Given the frequent use of interchangeable terms within the existing literature and the diverse approaches to pediatric palliative care systems globally, our research strategy was designed to encompass a broad spectrum of terminology. This included incorporating terms such as “palliative care,” “end-of-life care,” “terminal illnesses,” and “life-threatening illnesses” into our database search. By doing so, we aimed to address the variability in the terminology used by researchers and practitioners worldwide, ensuring that our review encompasses a comprehensive range of studies relevant to the care of children with serious or life-limiting conditions.

#### Inclusion and exclusion criteria

Inclusion and exclusion criteria were set at the project's outset (Table 1). Articles in English, focusing on pediatric palliative care settings’ physical environment and design within hospitals or hospices, were included; exclusion criteria covered adult care, dementia, healthcare quality assessment, pain experience, and illness progression.

**Table 1.** Criteria for Including and Excluding Materials.

Inclusion	Exclusion
Paper written in English	Papers describing the assessment of healthcare quality, the experience of pain, and the progression of illness
Papers examining end-of-life, terminally ill patients, life-limiting illness	Intensive care units
Papers examining end-of-life patients receiving ongoing palliative care for the palliative phase of their disease in inpatient units such as hospital, hospice, and nursing care	Papers focusing on pediatric intensive care units
Papers mentioning physical environment elements	Papers without a clearly defined methodology
Papers examines children and adolescents	Papers not focusing on the physical environment
No date limits	Review articles

#### Data extraction and analysis

After removing duplicates, the author (SN) screened titles and abstracts, applying predefined inclusion and exclusion criteria. Thematic analysis, utilizing NVIVO software, involved coding key excerpts and identifying themes from the selected references. The collaborative approach with authors (CS, RM) ensured robust findings, including multiple reviews of full texts for consensus on final inclusion, contributing to overall data interpretation.

## Results

**Figure 1.**
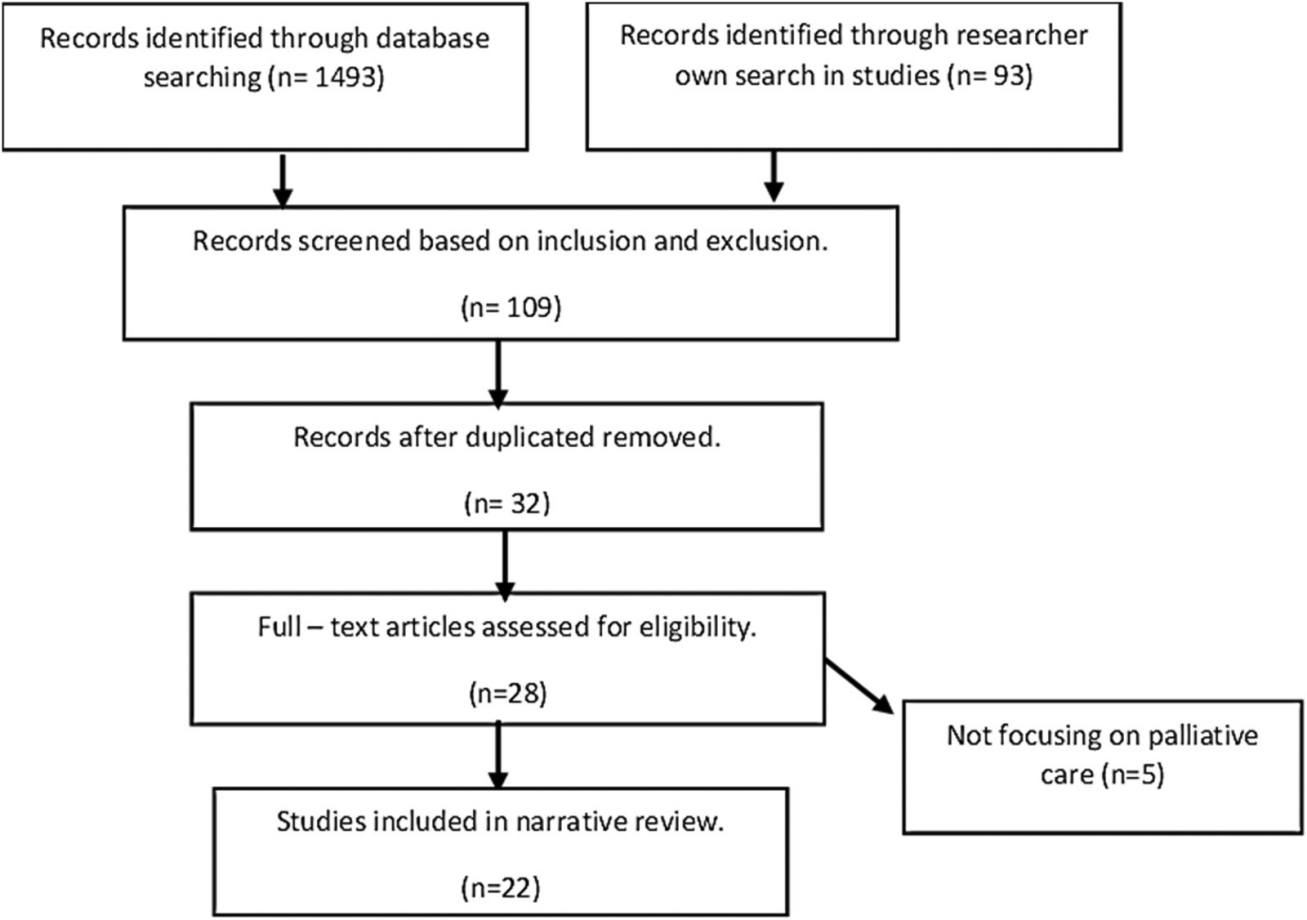
PRISMA flowchart.

In the initial database search, 1493 references were found, with an additional 93 collected independently. After screening, 109 references remained, and 77 duplicates were removed. Eventually, 28 studies meeting inclusion criteria were analyzed. Following consultation with all authors, five irrelevant references were eliminated, resulting in a final sample of 22 studies conducted across various countries, including the USA, UK, Canada, Australia, Italy, Netherlands, Iran, Indonesia, Japan, Kuwait, South Africa, and Uganda (Figure 1 and Table 2). Most of these papers focused on environments for terminally ill patients within hospital settings. Some of them were both in the hospital and hospice, and only six of them were in the hospice area. These studies emphasized environmental considerations in pediatric palliative care, highlighting four themes: the importance of activities and play environments, design considerations for accommodation spaces, supportive spaces for families, and outdoor and green spaces. Table 3 contains the summary of articles used in this narrative review.

PRISMA = preferred reporting items for systematic reviews and meta-analyses.

**Table 2.** Study Demographics (Authors).

Geographic region		Year of publication
Indonesia	*N* = 1	2020
UK	*N* = 7	2005, 2006, 2009, 2010 (*n* = 2), 2020 (*n* = 2)
Kuwait and UK	*N* = 1	2022
Australia	*N* = 3	2014, 2020, 2023
South Africa	*N* = 2	2014 (*n* = 2)
Canada	*N* = 1	2005
Italy	*N* = 1	2016
Japan	*N* = 2	2015, 2016
Iran	*N* = 2	2020, 2022
USA	*N* = 2	2005, 2009
Study design		
Qualitative study	*N* = 10	
Conceptual paper	*N* = 2	
Qualitative and quantitative study (mixed method)	*N* = 4	
Quantitative study	*N* = 2	
Descriptive paper	*N* = 1	
Qualitative exploratory study	*N* = 1	
Qualitative descriptive study	*N* = 2	

**Table 3.** Narrative Review Article Summary Used in This Paper (Authors).

Author(s) in Alphabetic Order and Year of Publication	Title	Aim/Objective of the Publication	Study Setting	Type of Publication	Research Method if Relevant	Key Findings/Points
Adistie et al. (2020)	The Needs of Children with Terminal Illness: A Qualitative Study	To investigate the requirements of children with terminal illnesses as perceived by both nurses and parents.	Setting: pediatric ward, pediatric intensive care unit (PICU), and neonatal intensive care unit (NICU) in hospital. Patient type: end-of-life or terminally ill patients or palliative.	Qualitative-descriptive study	Focus group discussion (FGD) with nurses and in-depth interviews with nurses and parents.	The significance of play, access to education, and robust social support networks from family, friends, and peers is underscored when addressing the social needs of individuals, particularly within their environment.
Aldiss et al. (2009)	What is Important to Young Children Who Have Cancer While in Hospital?	The objective of this study was to investigate the experiences and perspectives of children and young people regarding cancer care services.	Setting: inpatient ward of a hospital. Patient type: chronic illness (life threating/limiting illness). Limitation: recruiting children in palliative care was tough due to low caseloads.	Qualitative study	Interview with children and families. A lotto game with cards depicting different facial expressions.	The study emphasizes the importance of having playrooms located close to children's rooms within the hospital setting. Additionally, all children needed to have their parents stay with them during their hospitalization. Some children also expressed that bringing their toys from home helped make the hospital environment feel more familiar and comforting, akin to being at home.
Barling et al. (2014)	The Reality of Hospitalization: Stories from Family Members of their Hospital Experience for Adolescents and Young Adults Living with and Dying from Cancer	The primary aim of the major study was to explore the experiences of family members following the diagnosis, treatment, and eventual passing of an adolescent or young adult (AYA) aged 16–25 years.	Setting: inpatient ward of hospital. Patient type: end of life (died patients).	Qualitative study	Individual/face to face Interviews with family members.	The challenges encountered included the mismatch between the life stage of the AYAs and the environment of the hospital. The hospital wards were characterized as sterile and not conducive to healing.
Boucher et al. (2014)	The Role of Play in Children's Palliative Care	This paper explores the role and the value of play as an integral component in the provision of palliative care for children with chronic, life-threatening, and life-limiting conditions.	Setting: hospitals, outpatient clinics, inpatient care units, purpose-built children's hospices, community centers, and the child's own home. Patient type: end-of-life terminal illnesses or palliative.	Conceptual study	The study does not involve empirical research or data collection.	Highlighting the importance of providing appropriate play equipment, creating safe and supportive play environments, and establishing regular play times is crucial. It emphasizes the significance of trained adult supervision to facilitate therapeutic play experiences, which contribute to the psychosocial well-being of children facing chronic, life-threatening, and life-limiting conditions. Play serves as a means for communication, empowerment, and emotional support in this context.
Davies et al. (2005)	Children's Perspectives of a Paediatric Hospice Program	This study aims to evaluate the effects of the Canuck Place program, with a particular focus on its respite and end-of-life aspects, on both children with illnesses and their siblings.	Setting: hospice. Patient type: end-of-life or terminal illnesses or palliative.	Mixed method (qualitative and quantitative study)	Face-to-face interviews, survey based on qualitative findings from initial interviews.	Children expressed a preference for features such as the hot tub, school room, their rooms, special events, and interactions with peers, families, including siblings, and staff members. They appreciated the home-like atmosphere of Canuck Place and felt secure and well-cared for. While most siblings felt that Canuck Place was already ideal, older siblings suggested the incorporation of more activities geared toward teenagers. Child-oriented designs, such as images on bedroom walls and the use of vibrant colors, stimulated the imagination and contributed to a welcoming environment.
Davies (2005)	Mothers’ Stories of Loss: Their Need to be with their Dying Child and their Child's Body after Death	The initial objective of this study was to investigate the loss of a child from the perspective of parents.	Setting: hospital, hospice, intensive care units, home. Patient type: end-of-life or terminal illnesses or palliative.	Qualitative study	In-depth interviews with mothers.	The presence of private rooms enabled parents to customize the environment, establishing a space that was both comfortable and significant to them. They could spend meaningful time with their child's body after death, maintaining a sense of control over the space and privacy during these poignant moments.
Donnelly et al. (2005)	The Needs of Children with Life-limiting Conditions: A Healthcare-Provider-based Model	The objective of the current study was to develop a conceptual model based on empirical evidence that delineates the needs of children facing life-limiting conditions.	Setting: hospital, hospice, intensive care units, home. Patient type: End of life, terminally ill patients, palliative.	Conceptual paper	Concept mapping (which involves a structured process to organize and analyze complex data).	At the heart of the needs of children with life-limiting illnesses lies dignity and respect, with privacy serving as a pivotal component within this framework. In the concept map of the study, privacy emerges as the most central cluster, operating as a subset of dignity and respect.
Downing et al. (2014)	Children's Palliative Care: Considerations for a Physical Therapeutic Environment	The aim is to define children's palliative care, assess its recipients and need, emphasize the therapeutic environment, and offer insights on planning considerations for the physical environment in pediatric palliative care services.	Setting: hospital, hospice, home (not mentioned the exact setting as it is a descriptive study). Patient type: end-of-life or terminal illnesses or palliative.	Descriptive study	The study does not involve empirical research or data collection.	This study highlights the importance of creating a supportive and comfortable environment that fulfills children's psychosocial needs, including learning, play, and creative activities. This involves attention to outdoor areas and designing bedrooms to resemble a typical home, fostering a sense of safety and security. Proximity to family is crucial, and careful selection of materials, finishes, furnishings, color schemes, and lighting is necessary to create a welcoming, calming, and positive atmosphere. Additionally, the inclusion of play areas for siblings, private family spaces, areas for education, and flexibility in design to accommodate the diverse ages and individual needs of the children are essential considerations. Top of Form
Dunbar and Carter (2021)	Experiencing Place Identity and Place belongingness at a Children's Hospice: Parents’ Perspectives	This article demonstrates how parents’ views of the identity of the hospice change and how the hospice becomes a place where parents experience a sense of belongingness.	Setting: hospice. Patient type: end-of-life or terminal illnesses or palliative.	Qualitative study	Focus groups with parents of children with life-limiting conditions.	Parents emphasized the importance of a natural and unforced environment focused on keeping children happy. The hospice also provided support and activities for siblings. Encompassing rooms or special activity rooms to cater to the emotional and social needs of families about their child.
Dunbar et al. (2020)	Place Bonding’ in Children's Hospice Care: A Qualitative Study	The objective of this study was to investigate parents’ perspectives and experiences regarding hospice care, aiming to comprehend the barriers and facilitators to accessing hospice services, as well as identifying the characteristics parents desired from hospice provision.	Setting: hospice. Patient type: end-of-life or terminal illnesses or palliative.	Qualitative study	Focus groups and interview with parents to understand the barriers and/or facilitators to accessing a hospice.	Parents emphasize the importance of finding a place that feels like home for their child, highlighting the need for safety, familiarity, and a sense of belonging. Support in the form of presence near the child patient and offering appropriate activities can be for the entire family, including parents and siblings.
Gibson et al. (2010)	Children and Young People's Experiences of Cancer Care: Qualitative Research Study using Participatory Methods	The aim is to investigate children's and young people's perspectives on cancer care and to propose a conceptual model of communication and information sharing.	Setting: specialized hospital (cancer). Patient type: chronic illness (life threating/limiting illness). Limitation: recruiting children in palliative care was tough due to low caseloads.	Qualitative, exploratory study	Play and puppets, draw and write and activities day and interviews with patients in different stages of illness.	Creating a home-like environment involves fostering a non-medical atmosphere and incorporating items from home. The findings underscore the significance of acknowledging age-specific preferences and striking a balance between privacy and social interaction. Colorful decorations are also noted as important elements in the environment.
Gola et al. (2016)	Architectures for Paediatric Palliative Care: How to Improve Quality of Life and Environmental Well-being	The research aims to identify qualities that patients and their families regard as crucial elements, defining the social and architectural aspects of hospices.	Setting: hospital, hospice. Patient type: palliative or terminal illness.	Mixed method (qualitative and quantitative study)	Interview with experts and survey with healthcare professionals working in the field of palliative care.	Healing gardens and green spaces, user-centered design, usability of outdoor spaces, and flexible layout are key aspects emphasized in the research. The study suggests that pediatric palliative care facilities should ideally have fewer than ten beds and provide appropriate activity and play areas.
Ito et al. (2015)	Good Death for Children with Cancer: A Qualitative Study	The primary objective of this study is to investigate the attributes associated with a positive end-of-life experience for children with cancer.	Setting: hospital. Patient type: palliative or terminal illness or survivors of chronic illness.	Qualitative content analysis	In-depth interviews with the patient's survivor and bereaved family.	The outcomes of the study include the establishment of private spaces, access to educational, recreational, and playing activities, maintaining connections to usual activities and relationships from home, highlighting the significance of calming spaces, promoting family involvement, and providing accommodations for families to stay close to their children.
Jasem et al. (2023)	Caregivers’ Perspectives on the Social and Physical Environmental Factors Associated with the Play of their Children with Palliative Care Needs: A Q Methodology Study	The study aimed to investigate caregivers’ viewpoints on the crucial elements within the social and physical environments necessary for their children to engage in play within hospital or hospice settings.	Setting: hospital or hospice. Patient type: palliative, chronic illness (life threating/limiting illness).	Mixed method (qualitative and quantitative study)	Q methodology with caregivers.	Caregivers prioritize the presence of dedicated playrooms within healthcare facilities, offering spaces specifically designed for recreational activities and socializing among children. They emphasize the significance of upholding infection control measures within play areas to guarantee a safe environment. Moreover, ensuring that play areas and equipment are accessible to children with mobility challenges is also highlighted as crucial.
Kirk and Pritchard (2012)	An Exploration of Parents’ and Young People's Perspectives of Hospice Support	The objective of this study is to examine the perceptions of parents and young people regarding hospice support and to identify areas where support could be enhanced.	Setting: hospice. Patient type: palliative or terminal illness or end-of-life (dead patients).	Mixed method (qualitative and quantitative study)	Survey with parents and bereaved parents and interview with parents and young people.	Offering chances for children to connect with peers and participate in activities that promote social interaction is important. Additionally, siblings experience notable benefits when hospice activities are tailored to their specific age group.
McLaughlan and Pert (2020)	Briefing a Children's Hospice: Bridging the Evidence Gap and Redefining Value in Contemporary Healthcare Design	The objective of this study is to investigate evidence-based design principles for healthcare settings, with a specific focus on hospice and palliative care facilities.	Setting: hospice. Patient type: palliative or terminal illness or end-of-life (dead patients).	Qualitative study	Observation, photo-response interviews, and the draw your ideal with staff and interviews with staff and parents.	To enhance dignity and comfort, it is essential to expand bedroom sizes, provide individual ensuites, and address heating issues effectively. Additionally, incorporating adolescent-friendly spaces and establishing soft playrooms for active children are important considerations. Offering opportunities for engagement in gardens is beneficial for patients with low mobility. Furthermore, ensuring a private space for staff contributes to the overall quality of care.
Mitchell et al. (2006)	Care and Support Needs of Children and Young People with Cancer and their Parents	This study aims to depict caregivers’ experiences concerning the dignity of teenagers in the terminal stages of life.	Setting: treatment centers. Patient type: chronic illness (life threating/limiting illness).	Qualitative study	Postal survey with parents and children.	There is a need for additional age-appropriate toys and activities, particularly geared toward teenagers, as well as enhancements in car parking facilities. Parents also indicated a desire for a hospital library offering cancer-related books for borrowing. Furthermore, support for siblings and grandparents was generally perceived as insufficient.
Mohammadi et al. (2023)	Caregivers’ Perception of Teenagers’ Dignity in End-of-life Stages: A Phenomenological Study	The purpose of this study is to describe the caregivers’ experiences regarding teenagers’ dignity in the final stages of life	Setting: hospitals. Patient type: palliative or terminal illness or end-of-life (dead patients).	Qualitative study	Semi-structured interviews with caregivers.	The importance of preserving physical, psychological, and information privacy to maintain teenagers’ dignity. Need for same-sex caregivers.
Nagoya et al. (2017)	Paediatric Cancer Patients’ Important End-of-Life Issues, including Quality of Life: A Survey of Paediatric Oncologists and Nurses in Japan	The study aims to identify the key elements and constructive concepts of quality of life (QOL) at the end of life (EOL) for pediatric cancer patients.	Setting: hospitals. Patient type: palliative or terminal illness or end-of-life (dead patients).	Quantitative study	A cross-sectional survey with pediatricians and nurses resulting.	Engaging in play and learning activities, fulfilling wishes, and spending quality time with family are important aspects for pediatric patients. A peaceful death in the presence of loved ones is also valued.
Rollins (2009)	The Influence of Two Hospitals’ Designs and Policies on Social Interaction and Privacy as Coping Factors for Children with Cancer and their Families	The objective was to delineate how the physical attributes and protocols of a UK hospital and a US hospital supported coping mechanisms for children with cancer and their families.	Setting: hospitals. Patient type: chronic illness (life threating/limiting illness).	Qualitative study	Drawing and interviews, observation, and literature study. (The author reviewed findings from children's drawings, interviews, and observations, as well as photographs of hospital rooms and floor plans.)	This study contrasted hospital settings in the UK and USA, each characterized by distinct environmental and architectural features. The UK site featured an open-ward design, facilitating social interaction, and included a 24-h playroom. Conversely, the US site had single-occupancy rooms, promoting strong parent-to-child support but limited child-to-child interaction within the unit. Both sites acknowledged the significance of privacy. Colorful decorations and wall art were emphasized to enhance the overall ambiance and create a welcoming environment.
Smith et al. (2023)	Parents’ Experience of Extended Viewing in a Paediatric Hospice: A Qualitative Study	The study aimed to understand parents’ experiences of extended viewing after the death of a child with a life-limiting condition and to evaluate the quality of bereavement care provided in the first days following the death.	Setting: hospice. Patient type: palliative or terminal illness or end-of-life (dead patients).	Qualitative descriptive study	Semi-structured in-depth interviews with bereaved parents and children.	The parents found that the serene and calming ambiance of the cooled bedroom allowed them to cherish fond memories of their child's last moments in that space.
Zheng and Sedeh (2020)	Effect of Hospital Architecture, Computer Games, and Nurses’ Behaviour on the Effectiveness of the Treatment Process of Adolescent Cancer Patients	The study aims to propose a model for evaluating the impact of hospital architecture, computer games, and nurses’ behavior on the treatment effectiveness of adolescent cancer patients.	Setting: hospitals. Patient type: chronic illness (life threating/limiting illness).	Quantitative study	Questionnaire with descriptive statistics with adolescent cancer patients.	Highlighting the significance of technological interventions, particularly computer games, is crucial.

### Activities and Play Environments

Researchers have pinpointed essential environmental components for establishing responsive environments to pediatric patients’ needs in palliative care. One element underscored in several studies is the significance of playrooms, toys, and activities for pediatric patients in end-of-life medical settings (Adistie et al., 2020; Aldiss et al., 2009; Downing et al., 2014; Dunbar & Carter, 2021; Rollins, 2009). In the Children's Perspectives of a Pediatric Hospice Program study by Davies et al. (2005), children at a Canadian hospice expressed a desire for personal TVs, more activities, an abundance of toys, and improved access to these resources. Similarly, the studies by Boucher et al. (2014) and Ito et al. (2015), underscore the importance of play in the lives of children with life-limiting illnesses in healthcare settings. Boucher et al. (2014) emphasize not only the importance of playing spaces but also the need to respect children's right to play, communicate and provide specialized play therapy when necessary. Building upon this, Ito et al. (2015) highlight the positive impact of allowing pediatric cancer patients sufficient opportunities to play freely, emphasizing the role of unstructured play in their end-of-life experiences.

In the design of cancer treatment centers for adolescents, Zheng and Sedeh (2020) emphasize the importance of technological interventions, such as computer games. Jasem et al. (2023), in their study, explore caregivers’ perspectives on play for children with life-threatening conditions, underscoring the adaptation of activities to the child's abilities and the importance of infection control measures in play areas. Collectively, numerous studies consistently stress the crucial role of playrooms, toys, and activities in enhancing the well-being of pediatric patients in palliative care. Within the context of pediatric palliative care, essential elements for creating an effective and responsive play and activity environment include age-appropriate options, the continuous availability of familiar activities to cultivate a home-like atmosphere, and the promotion of social interaction through activities involving families and peers.

#### Age-appropriate activities and play

Studies indicate that children with chronic illnesses exhibit distinct preferences, particularly for age-appropriate and accessible information (Mitchel et al., 2006). This age-appropriate consideration extends to various aspects, encompassing information provision, activities, and leisure options for patients (Clark & Fasciano, 2015). Davies et al. (2005) were the first to report on age-appropriate playing and activities spaces in a hospice facility and concluded that there was a need for more extensive activities for both ill children and their siblings who express a desire for expanded activities tailored to older children and teens. They made the following suggestions for enhanced facilities, including advanced board games, video games, and age-appropriate movies. Furthermore, there is a call for more extensive amenities like larger basketball courts, volleyball courts, or even swimming pools to provide additional recreational options for older siblings. Mitchell et al. (2006) highlighted an unmet need for age-appropriate facilities and activities, particularly for teenagers, in healthcare settings catering to children and young people. This includes the provision of suitable toys, activities, and dedicated spaces for teenagers. A later study by Gola et al. (2016) agreed with the importance of catering to the distinct needs of adolescents by providing appropriate activity and play areas. Ensuring access to age-appropriate activities and facilities is important, not only for patients but also for supporting siblings who benefit significantly when hospice activities cater to their age group (Kirk & Pritchard, 2012). In the context of palliative care, Barling et al. (2014) noted challenges in catering to the unique needs of adolescents and young adults, including age-inappropriate activities.

#### Continued access to usual activities and plays

In a study by Aldiss et al. (2009), children appreciate having their familiar toys brought from home and personalizing their hospital space, creating a cosier and more home-like atmosphere. This action plays a crucial role in keeping the children engaged and preventing them from experiencing boredom during their hospital stay. In this study having access to routine activities and play helps provide the familiarity of their home environment. Ito et al. (2015) also discussed the importance of maintaining access to usual activities and relationships that children had in their own homes, in the palliative care settings.

#### Fostering social interaction through activities with families and peers

**Figure 2.**
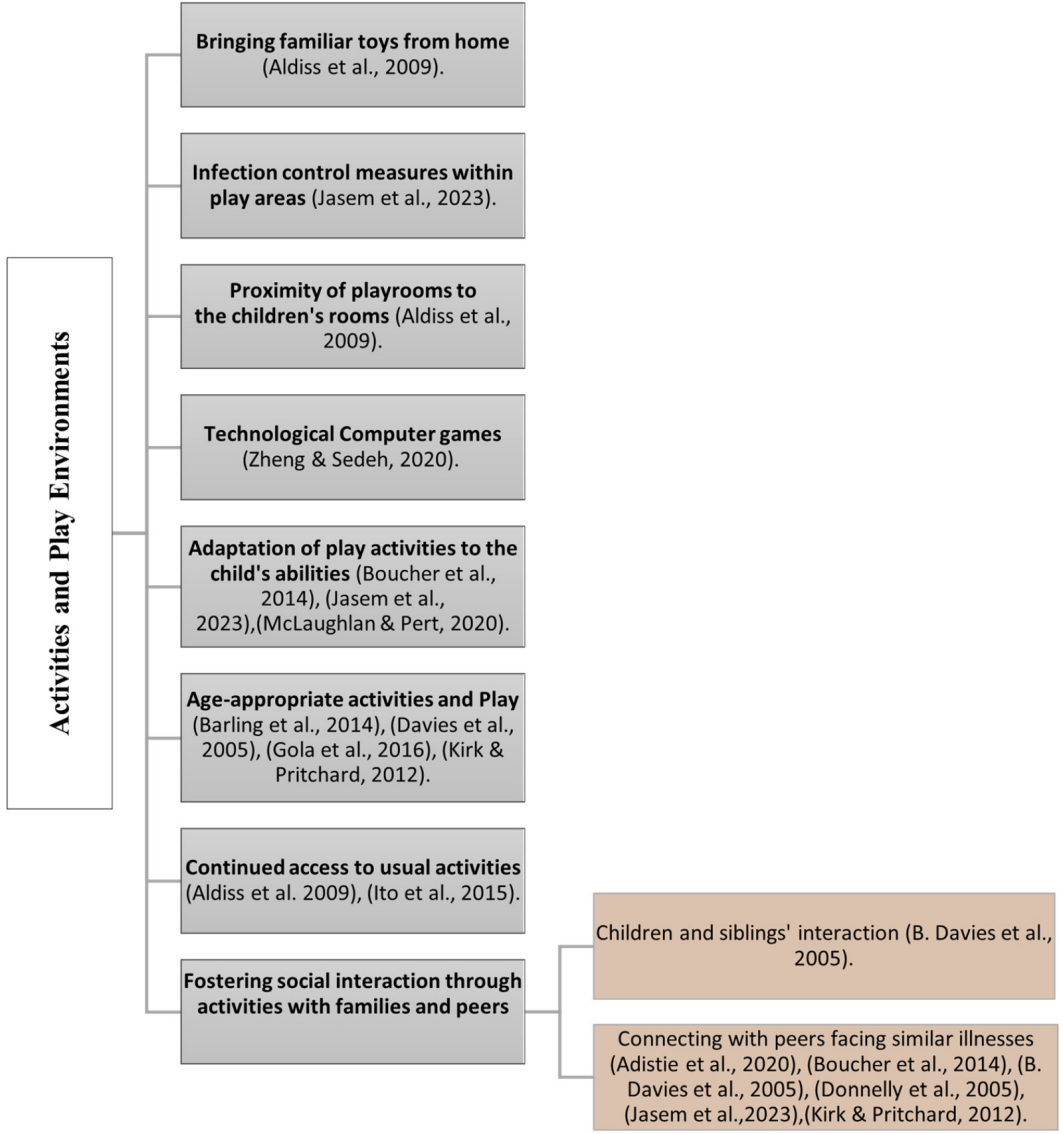
Activities and play environments in pediatric palliative care studies (authors).

Acknowledging the profound importance of social interactions in healthcare, numerous studies highlight how patients, particularly children, actively seek and benefit from social connections. This interaction often takes the form of play and activities involving loved ones, siblings, and peers who share similar conditions. At Canuck Place Hospice in Canada, both sick children and their siblings valued social interaction. The hospice's intentional design promoted connections in spaces like the kitchen, school room, dining room, and foosball/TV room, facilitating communication among peers with similar illnesses, their families, and staff (Davies et al., 2005). Donnelly et al. (2005) emphasized the effectiveness of assessing and promoting social interaction in a safe environment. Similarly, Kirk and Pritchard (2012) in their studies about parents’ views about hospice support, highlighted the importance of providing opportunities for children to meet peers and engage in activities for social interaction. Furthermore, in another study by Adistie et al. (2020), children with terminal illnesses express a fervent desire to be with loved ones, including family, siblings, and peers, as well as seeking support from other children facing similar challenges. Jasem et al.'s (2023) recent study underscores the importance of social interaction and shared play experiences, with a focus on concerns about the child's health condition (Figure 2).

### Design Considerations for Accommodation Spaces

#### Home-like environment

Families often prefer to take care of their children in the home setting (Noyes et al., 2022). Despite this preference, most pediatric deaths occur in hospitals (Australian Bureau of Statistics, 2021). Creating a home-like hospital environment is valuable for providing comfort and essential care. However, implementing such settings necessitates innovative approaches, as noted in some studies on pediatric palliative care. According to Davies et al. (2005, p. 256), both young patients and their siblings described the hospice as a “home away from home,” experiencing a keen sense of security. They valued the spaciousness, friendly faces, and calm atmosphere, noting the contrast with busier and less personal hospital settings. The consensus was that the facility felt more like a home than a hospital. In a study by Aldiss et al. (2009), some children were uncomfortable with hospital stays, and missed the familiarity of home and the presence of family, friends, and siblings. Interestingly, these children mentioned personalizing their hospital space by bringing toys from home for a cosier atmosphere. In this regard, the child's bedroom is a crucial space, designed to resemble a typical home bedroom to create a sense of safety and security (Downing et al., 2014). In Gibson et al.'s study (2013), children disliked certain aspects of the hospital environment, particularly the clinical appearance of the hospital bed. They expressed a desire for non-clinical environments in the caregiving setting and found ways to make the place more home-like, such as bringing toys for younger children or photographs of pets for adolescent patients. In another study to explore parents’ perspectives of a hospice for their child, the key findings revolve around the development of a theoretical concept termed place bonding. This highlights the importance of searching for a place that feels like home for their child, emphasizing the need for safety, familiarity, and a sense of belonging in the built environment of hospice (Dunbar et al., 2020). In McLaughlan and Pert's (2020) study, which focused on hospice facilities, chose carpeted floors and lounge chairs upholstered in regular fabric, avoiding hospital-grade vinyl. This decision aimed to maintain a home-like atmosphere, in line with their non-institutional approach. Balancing essential medical care with creating a home-like environment in an institutional setting poses a significant challenge, requiring a delicate equilibrium.

#### Age-appropriate spaces

Palliative care practices differ by age, with unique approaches for children, youth, and geriatric patients, resulting in varied family expectations in each age group (Abdel-Razek, 2022). Suggesting age-appropriate built environments for palliative care, Mitchell et al. (2006) emphasize the importance of separate accommodation, and kitchen facilities tailored to the specific age group, as highlighted by parents of children undergoing treatment. Later study by Gibson et al. (2010) agreed with the importance of catering to the distinct needs of adolescents as illustrated by the perspective of a 7-year-old child who complained about being in the same ward with younger children, “they (Babies) cry a lot, and it makes you feel nervous” (Gibson et al., 2010, p. 1401). Recognizing the diverse ages and individual needs in pediatric palliative care, flexibility in design is helpful. Downing et al. (2014) stress the importance of a flexible design approach to address age differences and prevent issues like young children feeling lost in adult-sized beds or adolescents feeling disrespected with child-sized bed assignments. McLaughlan and Pert (2020) highlight the importance of understanding adolescents’ preferences and creating dedicated spaces for them in a 12-bed hospice. This approach effectively contributes to designing a supportive environment for diverse age groups in palliative care.

#### Privacy

The studies of this review emphasize the critical role of privacy in pediatric healthcare settings. Privacy as a subset of dignity and respect forms the most central cluster in the concept map of a study by Donnelly et al. (2005). Rollins (2009) emphasizes privacy in child healthcare, noting the UK's use of dedicated activity rooms, the USA's patient room designs with curtains, and the provision of private lounges for parents with chairs, reading lamps, and phones. In Gibson et al.'s (2010) study, younger children show indifference to privacy, while older children prefer it. Jennifer, a 10-year-old patient, expressed frustration with compromised privacy, taking proactive measures like positioning her wheelchair behind the door. The research highlights challenges in open environments, including insufficient sound barriers and privacy concerns within cubicles. Curtains in the open setting failed to provide adequate sound barriers during private conversations. Extreme privacy, like medical isolation, posed challenges for younger children, leading to innovative connections, such as playing through windows via telephone. Pediatric cancer patients express the desire for private time and space, seeking moments alone with family and understanding from others (Ito et al., 2015). Gola et al.'s (2016) study suggests spacious rooms with fewer than ten beds and two semi-autonomous spaces totaling 40 m^2^ in pediatric palliative care facilities, equipped with meal preparation facilities and family gathering areas. Mohammadi et al.'s (2023) study emphasizes the importance of healthcare environments prioritizing physical, psychological, and informational privacy to maintain dignity in end-of-life care. The study also notes the preference of teenage patients for same-sex caregivers. Collectively, these studies highlight the crucial role of privacy in healthcare settings, addressing the needs of patients, their families, and healthcare staff.

#### Social interaction in wards

Rollins’ (2009) study compared UK and US hospitals and found that the UK case study site, with an open-ward design and a 24-h playroom, facilitated child-to-child interaction. In contrast, the US case study, with single-occupancy rooms, prioritized parent–child support but lacked opportunities for child-to-child interaction within the unit. The preference for having an open ward was highlighted in Gibson et al.'s (2010) study. It is noted that some older children preferred private rooms but acknowledged the benefits of open wards. Balancing beneficial social interaction with considering patient privacy, and infection control, which is a crucial factor, especially for patients in healthcare facility design seems to be challenging.

#### The visual sensory aspect of a place

**Figure 3.**
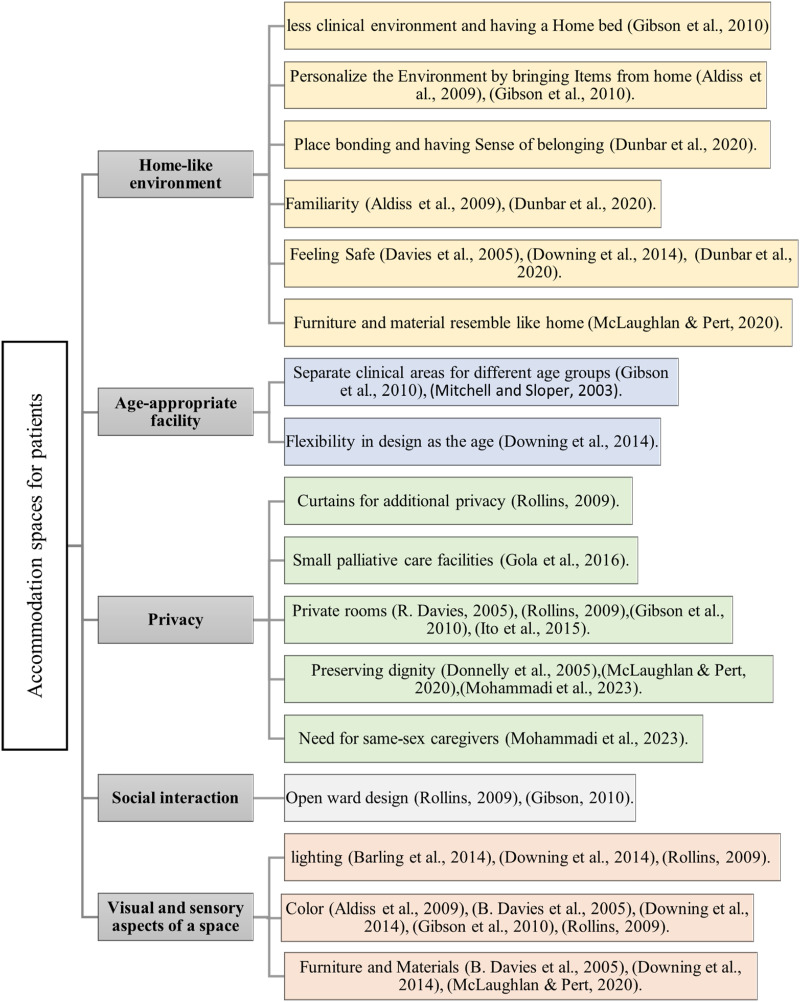
Accommodation spaces for patients in pediatric palliative care studies (authors).

Lighting, color, decoration, and materials can be also important in providing a responsive environment for pediatric patients. In 2005, Davies et al. emphasized the use of vibrant colors in hospice design to enhance childhood imagination and comfort, creating a more playful atmosphere. In this regard, Downing et al. (2014) stressed the importance of aligning color schemes with the intended purpose of each area, where specific colors promote relaxation, while others stimulate playfulness in children. Additionally, other studies (Adistie et al., 2009; Gibson et al., 2010; Rollins, 2009) also underscored the importance of colorful decorations and art on the walls to create a pleasant environment for children in the ward. Furthermore, natural light is effective in reducing depression and improving outcomes (Rollins, 2009). In one study, a dark environment was described as “scary and dodgy,” with an adolescent patient commenting that she “did not want to get better in a place like this” (Barling et al., 2014, p. 156). Downing et al. (2014) recommend incorporating strong natural light sources, such as large windows allowing direct sunlight. This, coupled with artificial illumination, fosters a cheerful atmosphere and enables children to perceive the day's distinct phases as the natural light changes throughout. In palliative care, decor, furnishings, and construction materials play a crucial role in the visual environment. In the Canuck Place program that specifically focused on pediatric hospice (Davies et al., 2005), child-oriented designs like images on bedroom walls fostered imagination. Rollins (2009) noted the pleasant impact of ceiling decorations and children's artwork in the ward. Downing et al. (2014) stress that material selection should prioritize resilience, durability, flexibility, and ease of cleaning to engage occupants’ intelligence and senses (Figure 3).

### Supportive Space for Families

**Figure 4.**
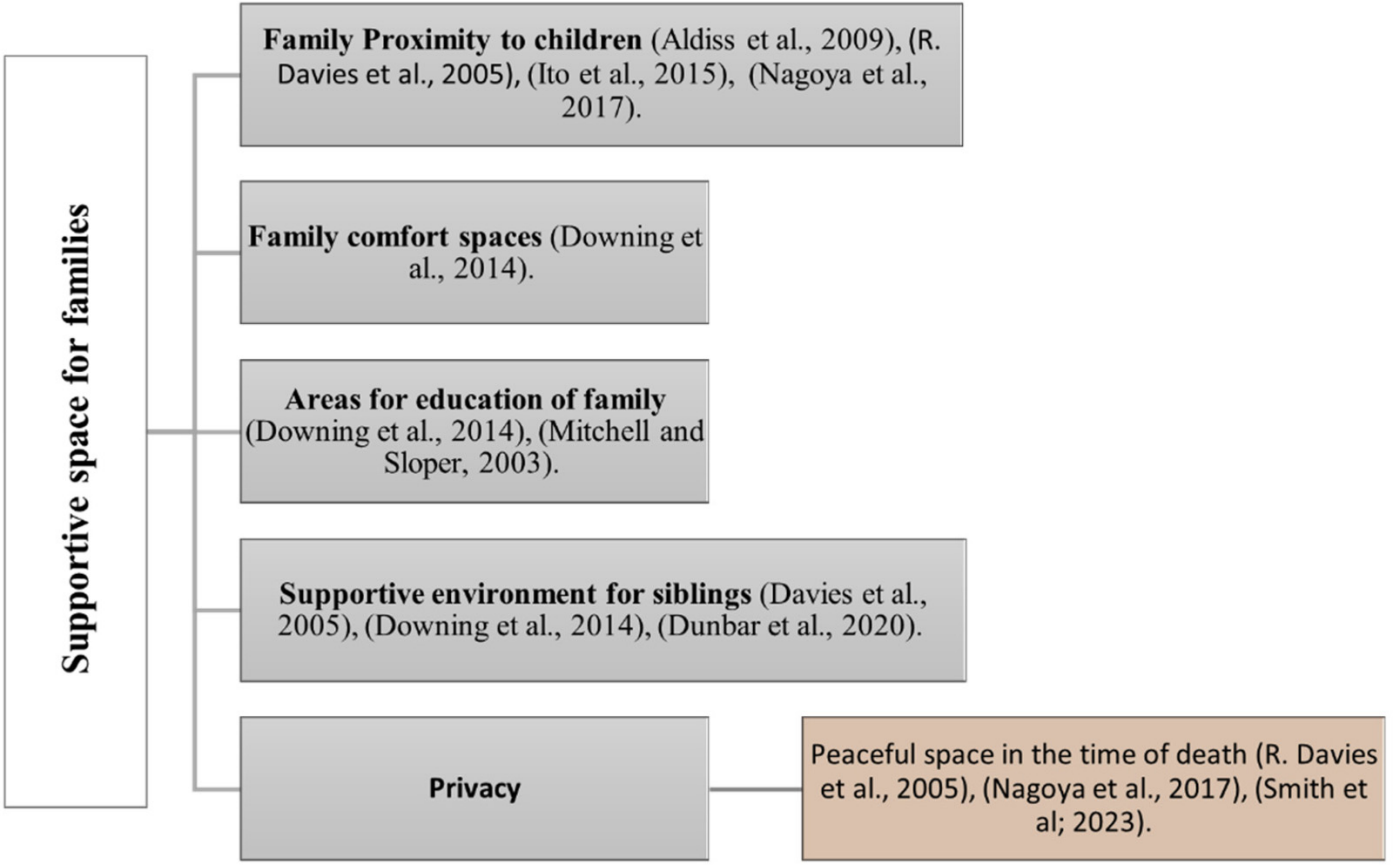
Supportive space for families in pediatric palliative care studies (authors).

Families of life-treating patients, often have unmet environmental needs that warrant consideration and attention. The healthcare environment can either promote collaborative relationships with parents as valued team members or create an unwelcoming atmosphere, reinforcing unequal relationships (Butler et al., 2019). In the context of children with cancer, parents staying with their hospitalized children are vital, as they are considered paramount in providing comfort and support (Aldiss et al., 2009). To facilitate this, dedicated space can be provided that allows families to stay close to their children (Davies, 2005; Downing et al., 2014; Ito et al., 2015). There are some other environmental needs for families that are mentioned in studies. These spaces should cater to various family needs such as educational zones like libraries (Downing et al., 2014; Mitchell et al., 2006), encompassing rooms or special activity rooms to cater to the emotional and social needs of families about their children (Downing et al., 2014; Dunbar & Carter, 2021). A significant need for families is the provision of an environment that enables peaceful moments with their loved ones during the patient's passing (Davies, 2005; Nagoya et al., 2017; Smith et al., 2023), such environment might allow families a private and personal bereavement process. Such support in the form of presence near the child patient and offering appropriate activities can be for the entire family, including parents and siblings (Davies et al., 2005; Downing et al., 2014; Dunbar & Carter, 2021). The findings underscore the critical significance of addressing families’ unmet environmental needs in pediatric palliative care. These needs include being close to patients, providing spaces for education, emotional and social support, and offering peaceful environments during the death (Figure 4).

### Outdoor and Green Spaces

**Figure 5.**
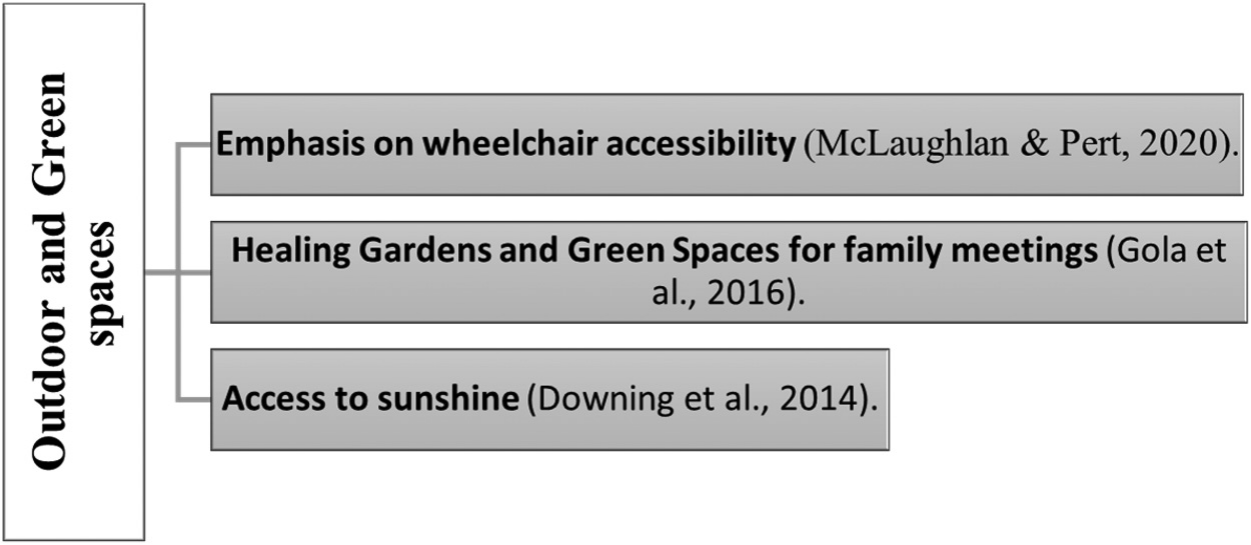
Outdoor and green spaces in pediatric palliative care studies (authors).

Consideration of outdoor areas and gardens for pediatric end-of-life patients is mentioned in palliative care studies as well. Designers of facilities can investigate methods to establish an attractive outdoor setting, allowing children, families, and staff to enjoy direct sunlight and promoting free movement in the open air (Downing et al., 2014). Green spaces, including healing gardens within the building, play a significant role in humanizing the care environment for children (Gola et al., 2016). The design of these outdoor spaces is meant to accommodate various age groups and their needs, making it a usable facility for families as well. The creation of healing gardens and green spaces for family meetings and training can be important as well (Gola et al., 2016). The importance of gardens and outdoor amenities in children's hospices was also mentioned by McLaughlan and Pert (2020), with an emphasis on wheelchair accessibility (Figure 5).

## Discussion 

The papers in this narrative review, many of which deal with information gathered from diverse inpatient units worldwide, collectively address shared concerns about the impact of the care environment on palliative or end-of-life patients and their families, exploring both positive and negative aspects. Each of the highlighted themes here touches upon specific points related to the quality of the respective environment feature. Despite some similarities, each theme presents unique nuances. We delve into a detailed discussion of these elements in the following sections.

One factor that warrants discussion here is the delicate balance between privacy and communal engagement within playing, accommodation, and family spaces in pediatric palliative care. Understanding the nuanced needs of patients, caregivers, and staff is crucial, as privacy requirements can vary significantly across different spaces and users. Among the studies examined, two emphasized patients’ desire to have private environments (Gibson et al., 2010; Ito et al., 2015), and even in pediatric patients, there are differences between age ranges. To illustrate this point, in a study by Gibson et al. (2010), it was found that younger children may struggle with excessive privacy, affecting their use of communal spaces, while older children typically prefer more private areas. However, some older children may still choose open wards in hospitals to stay connected with others. Parents, on the other hand, primarily emphasized the importance of a peaceful and private environment during their child's end-of-life moments, enabling them to come to terms with their child's passing (Davies, 2005; Nagoya et al., 2017; Smith et al., 2023). Experts, staff, and caregivers outlined other aspects of the environment that contribute to privacy, including considerations such as the size of rooms and the number of beds (Gola et al., 2016), ensuring private space for staff in the hospice facility (McLaughlan & Pert, 2020), and acknowledging the significance of privacy in creating spaces that preserve the dignity of teenagers, as observed by caregivers (Mohammadi et al., 2023). It is worth mentioning that most studies surveyed do not quantify what is meant by privacy exactly, highlighting the need to consider its meaning and features in the context of palliative care.

Although the provision of privacy is deemed important, some studies also underscore the need for social interactions, creating a notable contrast in this context. Children and young people have emphasized the significance of communication with their peers which can be done in playing environments in studies by Davies et al. (2005) and Kirk and Pritchard (2012) who both focused on hospice environments. This is also achievable by having multi-bed accommodation (Gibson et al., 2010). It is not only about interacting with peers, connections with family members including siblings, are also crucial (Adistie et al., 2020).  It is important to acknowledge that these connections must be evaluated within the context of the communication challenges arising from compromised immunity in such circumstances. In this context, it is essential to provide both single-patient rooms and shared rooms (Brereton et al., 2012). Single rooms are necessary for patients with distressing symptoms, but the option of having shared rooms should always be available for patients who prefer to have company (Kirk, 2002), a consideration that can be extended to pediatric patients as well. 

Another significant factor worth exploring within the themes of pediatric palliative care is the emphasis on a home-like environment. Research indicates that patients and families often express a preference for home-based care due to its perceived advantages in terms of privacy and freedom (Noyes et al., 2022). Moreover, findings suggest that cultivating a home-like atmosphere, particularly through smaller care settings and allowing for the incorporation of personal items from home, can greatly enhance the comfort of pediatric patients and their families. This sentiment is echoed in pediatric palliative care recommendations from Gola et al. (2016) advocates for smaller units with dedicated spaces for various activities, aligning with the notion of personalizing the environment through items from home, as supported by Aldiss et al. (2009) and Gibson et al. (2010) in pediatric studies. Despite the well-intentioned emphasis on creating home-like environments, further investigation is warranted to determine whether pediatric patients perceive these settings as comforting and if they hold significance in their care experiences.

Introducing another salient aspect within the discourse of pediatric palliative care, we turn our attention to a somewhat understated yet crucial element and this is the importance of maintaining links to familiar routines, play, and activities. This entails ensuring patients can partake in activities they enjoy and have spaces conducive to play and social interaction. As mentioned by Joseph et al. (2008), maintaining these connections is crucial for helping children adjust to the challenges of hospitalization. Kassam et al. (2014) emphasize that home is the place where a sense of normality is most strongly felt. Engaging in home-like everyday activities has been shown to generate a sense of home for palliative care patients at hospitals, as noted in research by Collier et al. (2015). This is always going to be a complex undertaking, given the diversity of patients and their homelives, that come to find themselves in a singular palliative care facility. Providing opportunities for hospitalized children to interact with their peers may be one way to help achieve this goal (Rollins, 2009). Ito et al. (2015) explored the idea of school interactions and forming friendships in a hospital setting as a positive aspect of end-of-life care for children with cancer. According to Fels et al. (2001), interaction with peers among pediatric patients yields significant benefits, including enhanced social and communication skills, as well as the development of self-confidence and independence. Additionally, Taylor et al. (2021) emphasized the significance of providing personal technologies, like internet access, for young patients during their stay away from home. Technological devices not only facilitate connection with friends but also act as a distraction when a child might experience feelings of loneliness or sadness. Donnelly et al. (2005) emphasize that children with life-limiting illnesses necessitate continuity of normalcy within their family, school, and faith/social community to address their needs. The findings highlight the importance of enabling pediatric patients to engage in familiar activities like school participation, peer interaction, and access to personal technologies, fostering familiarity and supporting routine activities.

### Limitation 

Due to limited research in the environmental and architectural aspects of pediatric palliative care, the review focuses on studies that extend beyond architecture. Notably, the majority of data stems from the perspectives of families and healthcare staff, revealing a significant limitation as patient preferences are not extensively explored (Levine et al., 2017). The included studies predominantly rely on the viewpoints of families and/or caregivers (Barling et al., 2014; Davies, 2005; Dunbar et al., 2020; Dunbar & Carter, 2021; Jasem et al., 2023; Mohammadi et al., 2023; Smith et al., 2023), families and staff (Adistie et al., 2020; Kirk & Pritchard, 2012; McLaughlan & Pert, 2020), and exclusively staff (Donnelly et al., 2005; Gola et al., 2016; Nagoya et al., 2017). A limited number involve both families and patients (Aldiss et al., 2009; Davies et al., 2005; Mitchell et al., 2006), with only one encompassing the perspectives of families, staff, and patients (Ito et al., 2015). Notably, three studies focus solely on patients’ viewpoints (Gibson et al., 2010; Rollins, 2009; Zheng & Sedeh, 2020), and two concentrate on existing literature, theories, and concepts (Boucher et al., 2014; Downing et al., 2014).

The limited availability of visual materials in the selected studies (only two out of 22 provided diagrams or pictures) prevented us from creating a schematic diagram to illustrate design environments. This shortage highlights gaps in architectural studies regarding guidelines for pediatric palliative care settings. Another limitation is that so many of the terms and descriptors used to characterize environments in this select body of literature tend to be generalized and more vague than architectural literature itself might be. Additionally, the variability in palliative care based on patient age led to grouping children and youth as a pediatric category due to limited research in more specific age groups.

## Conclusion

The environmental design of pediatric palliative care demands significant enhancements to cater to the diverse physical and spiritual needs of patients and their families. This narrative review identifies four pivotal environmental considerations: activities and play environments, design considerations for accommodation spaces, supportive spaces for families, and outdoor and green spaces. Notably, tailoring amenities to different age groups, incorporating personalized items from home, and creating a less clinical, home-like atmosphere are key aspects of building a supportive environment. Family support remains crucial, emphasizing comfortable spaces during the child's passing and addressing the needs of siblings. Balancing privacy and social interaction through open-ward designs for peer communication and single-occupancy rooms for prioritizing family relationships appears effective. However, the review points out notable research gaps, including the absence of studies from the patient's viewpoint and a lack of dedicated research on the environmental needs of adolescent end-of-life patients. Future research should address these gaps, exploring privacy factors, understanding the concept of a home-like environment, and delving into the unique perspectives of adolescent patients.

## Implications for Practice

- The creation of an activity and playing environment is important in pediatric palliative care settings, considering age-appropriate playing settings, ensuring access to routine activities, and fostering social interaction with both families and peers.
- Designing accommodation spaces with a home-like environment, age-appropriate spaces, privacy considerations, and attention to visual sensory aspects is crucial for creating supportive environments for pediatric patients in palliative care.
- The provision of supportive spaces for families of pediatric patients facing life-threatening conditions is essential, addressing their unmet environmental needs and facilitating proximity, emotional support, and peaceful moments during the patient's passing.
- Outdoor and green spaces, like healing gardens, in pediatric palliative care aim to offer natural sunlight and outdoor activities, fostering a supportive environment for families and staff.
